# COVA (cardiac output valve area): a reliable method for determining aortic transvalvular pressure gradients that does not use phase contrast imaging

**DOI:** 10.1186/1532-429X-16-S1-P247

**Published:** 2014-01-16

**Authors:** Kunal Patel, Seth Uretsky, Sunil Penesetti, Kavya Jain, Steven Wolff

**Affiliations:** 1 St. Luke's and Roosevelt Hospitals, New York, New York, USA; 2 Carnegie Hill Radiology, New York, New York, USA

## Background

Echocardiography is often used to quantify the transvalvular pressure gradient in patients with aortic stenosis. Velocity-encoded MRI is alternative method for quantifying the aortic transvalvular pressure gradient. However, the acquisition of additional phase-contrast images is time consuming and the analysis can be technically difficult. We report on the results from a new method, Cardiac Output Valve Area (COVA), for quantifying the aortic transvalvular gradient that does not rely on phase contrast images. Instead the instantaneous pressure gradient is computed from the cardiac output (based on the frame-by-frame changes in left ventricular systolic volume) and valve area. The purpose of this study is to compare the results from all three methods.

## Methods

This retrospective study is comprised of 102 consecutive patients who were referred for cardiac MRI and had aortic stenosis (72 ± 11 yrs, 64% male). Exclusion criteria included an irregular cardiac rhythm, a prosthetic aortic valve, or more than minimal mitral regurgitation (regurgitant volume > 10 ml). COVA mean and peak pressure gradients were determined using SuiteHeart software (research version, Neosoft, Pewaukee, WI). For inputs, the software required multiphase segmentation of the left ventricular endocardium and planimetry of the aortic valve. The results are compared to those obtained from velocity-encoded MRI (using the modified Bernoulli equations: peak pressure gradient = 4*v2, mean pressure gradient = 2.4*v2), and the clinical results from Doppler echocardiography (n = 24). To assess interobserver variability of the COVA method, a subset of 20 patients were analyzed by 2 blinded readers.

## Results

**Figure 1:**
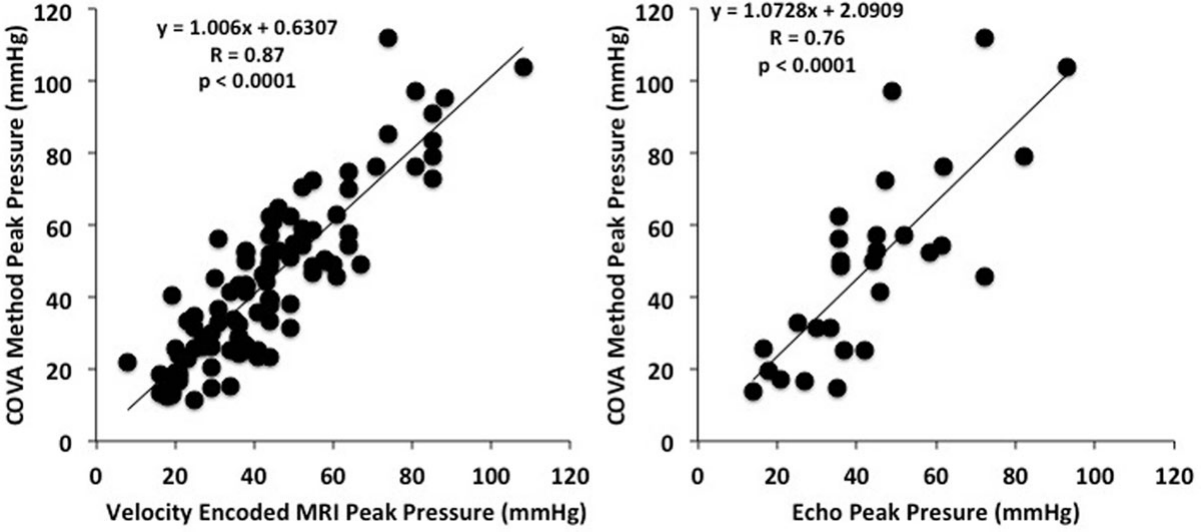
**Pearson's correlation comparing the peak pressure calculated using the COVA method to velocity encoded MRI and Doppler Echocardiography**.

Among the 102 patients with aortic stenosis, 34 (33%) were mild, 50 (49%) were moderate, and 18 (18%) were severe. There was a good correlation between the peak gradients as assessed by COVA and velocity-encoded MRI (r = 0.9, p < 0.0001) and between COVA and echocardiography (r = 0.8, p < 0.0001) (Figure 1). There was a good correlation between the mean gradients as assessed by COVA and velocity-encoded MRI (r = 0.8, p < 0.0001) and between COVA and echocardiography (r = 0.6, p < 0.0001). When characterizing the degrees of aortic stenosis as mild, moderate or severe, the agreement between COVA and velocity encoded MRI was 79%, and between COVA and echocardiography was 83%. There was a high degree of reproducibility for the COVA method between observers for the peak pressure (ICC = 0.92, 95% CI 0.73-0.97, p < 0.0001) and the mean pressure (ICC = 0.92, 95% CI 0.73-0.97, p < 0.0001).

## Conclusions

The aortic transvalvular pressure gradient as assessed by COVA correlates well with other accepted measures for assessing AS severity such as velocity encoded MRI and echocardiography. This method does not require the acquisition or analysis of phase-contrast images. In principle, it can be applied to other modalities such as cardiac CT.

## Funding

None.

